# The single point insulin sensitivity estimator (SPISE) is associated with bone health in Arab adults

**DOI:** 10.1007/s40520-024-02789-5

**Published:** 2024-06-21

**Authors:** Nasser M. Al-Daghri, Kaiser Wani, Malak N. K. Khattak, Abdullah M. Alnaami, Yousef Al-Saleh, Shaun Sabico

**Affiliations:** 1 Biochemistry Department, College of Science, King Saud University, Riyadh, 11451 Saudi Arabia; 2 Department of Medicine, Health Oasis Hospital, Riyadh, Saudi Arabia

**Keywords:** Hyperglycemia, Bone mineral density, SPISE, Type 2 diabetes mellitus, Insulin resistance, Osteoporosis

## Abstract

**Background:**

The Single Point Insulin Sensitivity Estimator (SPISE) index is a surrogate marker for insulin sensitivity. Given the emerging role of bone as an active endocrine organ, its associations with non-invasive measures of extra-skeletal functions such as insulin sensitivity warrant investigation.

**Aims:**

This study aimed to explore the relationship between the SPISE index and Bone Mineral Density (BMD) in an adult population.

**Methods:**

Data from a total of 1270 Arab adults (84% females, mean age 56.7 ± 8.1 years) from the Osteoporosis Registry Database of the Chair for Biomarkers of Chronic Diseases in King Saud University, Riyadh, Saudi Arabia was used in this study. T-scores and SPISE were calculated. Regression models were used to determine associations between SPISE and bone health indices.

**Results:**

The low BMD group (*N* = 853; T-score <-1.0) had significantly higher SPISE values than those with normal BMD (*N* = 417; T-score − 1.0 and above) (4.6 ± 1.3 vs. 4.3 ± 1.2, *p* < 0.001). Multivariate linear regression, adjusted for covariates, confirmed a significant inverse association between SPISE and BMD for all participants (β=-0.22, *p* < 0.001), as well as both groups [normal BMD (β = -0.10, *p* = 0.02) and low BMD groups (β = -0.15, *p* < 0.001)]. SPISE, family history of T2DM, and history of fractures collectively account for 17% of the variances perceived in T-score for all participants (*p* < 0.001).

**Conclusions:**

A significant inverse association between the SPISE index and BMD was observed in adults, suggesting a link between BMD and extra-skeletal health. Underlying mechanisms need to be investigated prospectively using BMD as secondary outcomes in lifestyle modification programs.

**Supplementary Information:**

The online version contains supplementary material available at 10.1007/s40520-024-02789-5.

## Introduction

Osteopenia (T-scores between − 1.0 and − 2.5) and osteoporosis (T-score < -2.5) are conditions associated with low bone mass density (BMD) and increasing age which elevates risk for bone fractures, morbidity, mortality and incapacity [1, 2]. An estimated 200 million people suffer from osteoporosis worldwide, with an estimated prevalence of 1–8% in men and about 9–38% in women in developing countries [3, 4]. Saudi Arabia is no exception in terms of having a high prevalence of osteoporosis among adults > 60 years, with a community-based study from Riyadh (*N* = 1302) reporting 8.2% and 11.8% overall prevalence at femoral and lumbar sites, respectively [5]. In a recent study, 17,425 osteoporosis-related fractures were recorded in Saudi Arabia, with a total direct healthcare cost reaching USD 636 million [6].

Type 2 diabetes mellitus (T2DM) is also a highly prevalent chronic condition in Saudi Arabia [7]. Consequently, T2DM has also been associated, though inconsistently, with changes in bone microarchitecture [8, 9]. T2DM has been linked with an increased incidence of fractures [10, 11] and reduced BMD with poor glycemic control [12, 13]. Nevertheless, a recent meta-analysis of 14 studies of 24 340 participants concluded no relationship between T2DM and low BMD [14]. It is worth noting that despite the large sample size of the meta-analysis, most studies included were considered of low-quality evidence, having different sites and techniques used which were technically not comparable.

A simple novel surrogate marker to assess the insulin sensitivity, the Single Point Insulin Sensitivity Estimator (SPISE), is a non-invasive, easily calculated measure based on body mass index (BMI), HDL-cholesterol, and triglycerides [15]. A distinct advantage of this index lack of huge variability found in insulin assays [16], which is used to calculate traditional indices for insulin resistance (IR) like homeostasis model assessment for insulin resistance (HOMA-IR), the quantitative insulin sensitivity check index (QUICKI) and the McAuley index. This index has shown comparable sensitivity and specificity with the gold standard measure for insulin resistance (euglycemic clamp method) [17–19], which, compared to SPISE, is more complex, resource-intensive and not suitable for research studies. Furthermore, the SPISE index has also been associated with various metabolic disorders, including dyslipidemia, hypertension, and cardiovascular diseases [20].

Given that HOMA-IR and insulin sensitivity were observed to alter BMD [21, 22], and that the bone tissue itself has been implicated as an active endocrine organ that influences various metabolic processes, including glucose homeostasis [23, 24], there is reason to believe that a cross-talk exists between skeletal and endocrine systems. While previous research has established associations between HOMA-IR and bone health, our study addresses a critical gap by focusing on middle-aged to elderly Arab adults, an underrepresented demographic in bone health research. Besides, the novel SPISE index may serve as a practical and relevant tool to assess metabolic health status and its potential impact on BMD. Hence, the present observational study aimed to investigate the association between T-Score (spine) and SPISE index in middle to old-aged Saudi adults to examine the potential clinical utility of this novel SPISE index in predicting bone health outcomes. To the best of our knowledge, this is the first large-scale study in the Middle East to investigate a possible association between low BMD with HOMA-IR using the SPISE index.

## Materials and methods

In this cross-sectional study, the clinical information of participants was taken from the Osteoporosis Registry Database of the Chair for Biomarkers for Chronic Diseases (CBCD), a collaborative project of King Saud University (KSU) and the Ministry of Health [25]. In brief, the osteoporosis registry database contains clinical information of all Saudi adults whose BMD was assessed in several tertiary hospitals in Riyadh [King Fahad Medical City (KFMC), King Khalid University Hospital (KKUH), and King Salman Hospital (KSH)] from 2013 to 2016 [26–28]. The investigators adhered to all applicable ethical standards for collecting, storing, and analyzing human samples. Before being included in the study, all participants provided written informed consent. Ethical approval was obtained from the Institutional Review Board (IRB) of the College of Medicine in KSU, Riyadh, Saudi Arabia.

### Study design and participants

**Fig. 1:**
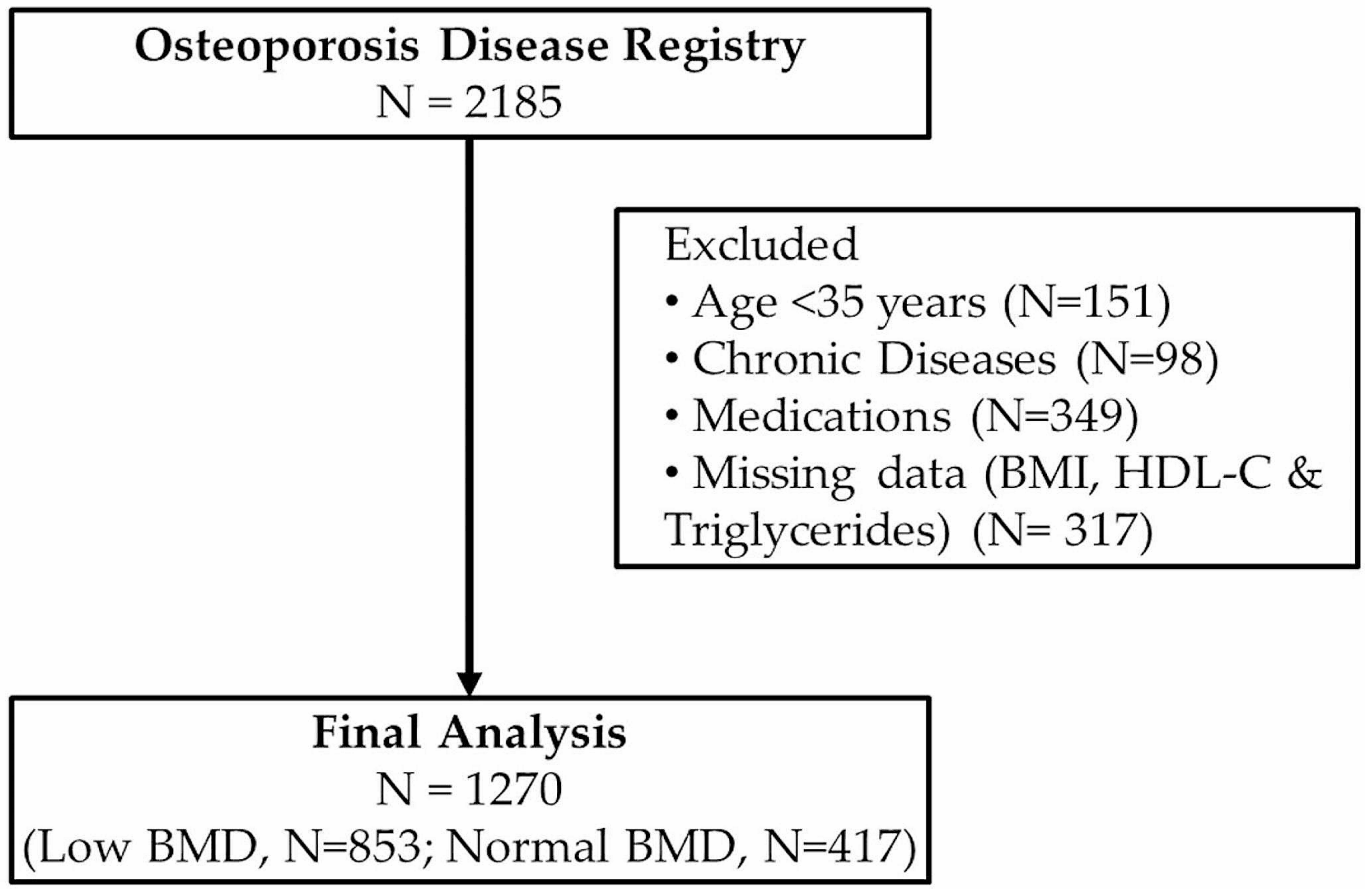
Flow chart of the selection of the study population from the Osteoporosis Disease Registry

For this study, samples from a total of 1270 participants (84% females) were used from the Osteoporosis registry after excluding the participants below 35 years of age, those with known malignancies and heart disorders, under medications that affect both BMD and glycemic status such as glucocorticoids, and those with missing biochemical information, necessary for SPISE calculation. Demographic information including age, sex, medical history and risk factors associated with bone loss (e.g., family history of osteoporosis, diabetes, or arthritis, thyroid disease, scoliosis of the spine, kyphosis, loss of height in last two years, had a fracture in the last five years) were noted. Figure 1 provides a flowchart of the study population.

### Bone mineral density (BMD) scan, T-Score and the study groups

Bone mineral density (BMD) at lumbar vertebrae L1–L4 was measured using a dual-energy X-ray absorptiometry (DXA) machine (Hologic Inc., Marlborough, MA, USA) in all the participants, and T-scores were obtained. The apparatus was checked for accuracy using a standard phantom that the manufacturer supplied, and a certified bone densitometry technician carried out the test. After being printed, the findings of the bone density scan were taken to the CBCD at KSU to have the data entered. The T-scores were used to categorize the individuals into ones with low BMD (T-score < -1.0) and normal BMD (T-score of -1.0 and above), the cut-offs of which were recommended by the World Health Organization [29] and used by the national and regional guidelines to identify the individuals at risk for bone loss [30–32].

### Sample collection, anthropometric and biochemical evaluations

Information of included participants on anthropometric evaluation (body mass index, BMI; waist and hip circumference and blood pressure) and biochemical results (glucose and lipid profile) were retrieved from the registry. In brief, height (cm) and weight (kg) were determined using a standard scale equipped with an affixed stadiometer and BMI (kg/m^2^) was calculated to determine overweight (25.1–29.9 kg/m²) and obese (≥ 30.0 kg/m²) participants. The circumferences of the waist (cm) and hips (cm) were assessed using a standard tape measure. Systolic and diastolic blood pressure (mmHg) were measured twice with 15 min interval while a seated position using an automated instrument (Omron-705CP; Omron Corp., Tokyo, Japan). All fasting blood samples were assessed and stored in the Chair for Biomarkers of Chronic Diseases (CBCD) at King Saud University (KSU), Riyadh, KSA. Fasting glucose, total cholesterol, HDL-cholesterol, triglycerides, calcium, and albumin were measured routinely with a biochemistry analyzer (Konelab 20XT, Thermo Scientific, Vantaa, Finland) [33] as done previously.

### SPISE index calculation

SPISE index was calculated using BMI and HDL-cholesterol (converted to mg/dl using factor 38.67) and triglycerides (converted to mg/dl using factor 88.57) as follows:

*SPISE index = (600 × (HDL − cholesterol in mg/dL)*^*0.185*^*)/ (Triglycerides in mg/dL)*^*0.2*^*× (BMI in kg/m2)*^*1.338*^ [15].

The SPISE index cut-off of 6.61 corresponds to the glucose disposal rate (M-value) of 4.7 mg/kg/min and lower than this cut-off indicates IR with an area under the curve of 0.797, the sensitivity of 66% and specificity of 80% [15].

### Data analysis

The sample size was calculated by the G*Power online calculator. For studying mean differences between two independent groups, a modest effect size of 0.5, a 5% tolerable error of α, and a desired power of 95%, we needed a total of 105 participantss in the two study groups. In total the osteoporosis registry contains clinical information of 2185 adults. SPSS version 28.0 (SPSS, Inc., Chicago, IL, USA) was used to analyze the data. We used the Kolmogorov-Smirnov test to ensure that our data were normally distributed. Normally distributed data were presented as mean and standard deviation (SD). Non-normal data were presented as median (1st and 3rd quartiles). Categorical variables were shown as frequencies (percentages). Bivariate associations were determined using Pearson’s and Spearman’s correlations for normally and non-normally distributed variables. Data was divided into two groups: ones with normal BMD and low BMD to assess the association in individual groups. Differences between these groups were compared using the Kruskal-Wallis H test and the one-way analysis of variance (ANOVA). Multivariate linear regression was then performed using T-score (spine) as a dependent variable and SPISE as an independent variable. Model “a” was adjusted for age, model “b” was adjusted for age and family history of T2DM, model “c” was adjusted for age, family history of T2DM, menopausal status in females, lost height in the past 2 years, and history of fracture in the last 5 years. A stepwise regression analysis was also conducted to investigate significant predictors for T-score (spine). Statistical significance was set as *p* < 0.05. MS Excel 2010 was used to prepare all the figures.

## Results

### General characteristics of participants

**Fig. 2:**
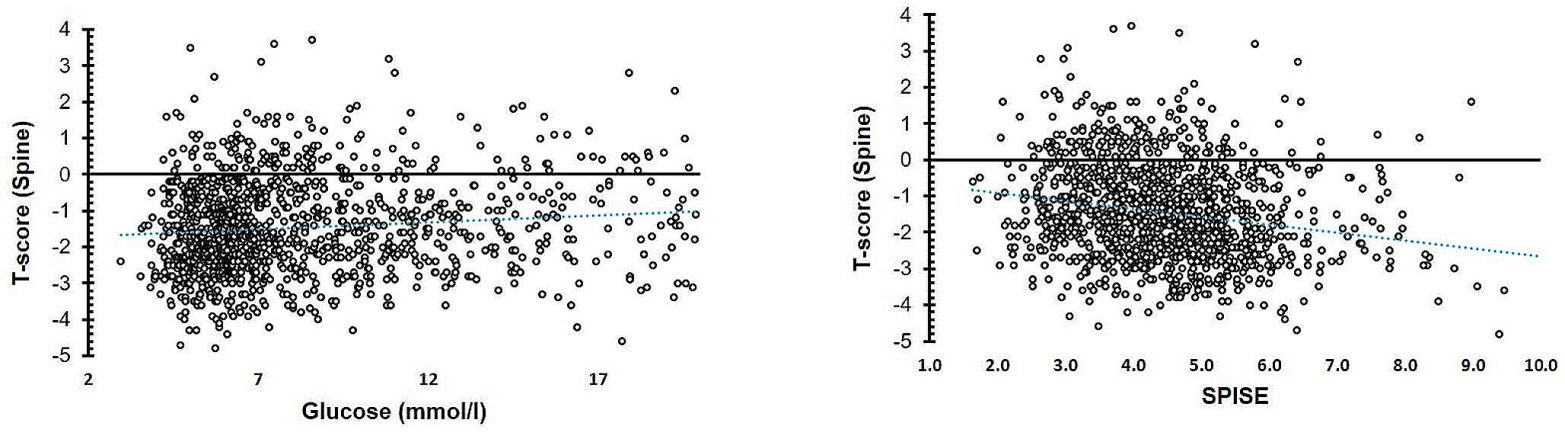
Bivariate associations of lumbar T-score with **A**) fasting glucose levels and **b**) SPISE index values in all participants

A total of 1270 Saudi adults (84% females; mean age 56.7 ± 8.1 years) were included in this study. The general characteristics are presented in Table 1. 91.6% of females were post-menopausal. Around 78% of participants were above 50 years old (*N* = 991). Around 91.9% (*N* = 1167) were either overweight or obese. A total of *N* = 417 (32.8%) had normal BMD levels and *N* = 853 (67.2%) had low BMD levels. Compared with the normal BMD group, the low BMD group had more females (89.2% vs. 73.4%, *p* < 0.001), more participants aged > 50 years (84.2% vs. 65.5%, *p* < 0.001) and less with family history of T2DM (56.7% vs. 71.2%, *p* < 0.001). Those with low BMD had significantly lower levels of fasting glucose and triglycerides (p-values of 0.002 and < 0.001, respectively) than those with normal BMD. The SPISE value was significantly higher in the low BMD group than the normal BMD group (4.6 ± 1.3 vs. 4.3 ± 1.2, *p* < 0.001). The distributions of T-scores and SPISE in males and females, and in pre and post-menopausal females were provided as histograms in supplementary figures S1 and S2 respectively. The bivariate association of BMD with fasting glucose levels and SPISE index values were presented as scatterplots in Fig. 2.

**Table 1:** General and anthropometric characteristics of participants divided according to the BMD (spine)

	All	Normal BMD	Low BMD	*p*-value
*N*	1270	417	853	-
T-score (L1-L4 Spine)	-1.46 ± 1.3	0.03 ± 0.8	-2.18 ± 0.8	< 0.001
Female	1067 (84.01)	306 (73.4)	761 (89.2)	< 0.001
Menopause^a^	977 (91.6)	246 (80.4)	731 (96.1)	< 0.001
**Anthropometrics**				
Age (years)	56.7 ± 8.1	54.1 ± 8.9	58.0 ± 7.4	< 0.001
BMI (kg/m^2^)	32.3 ± 6	33.0 ± 6.1	32.0 ± 6.0	0.004
Overweight Obese	377 (29.7) 790 (62.2)	111 (26.6) 277 (66.4)	266 (31.2) 513 (60.1)	0.09
Waist (cm)	100.2 ± 14.3	103.0 ± 14.2	98.8 ± 14.1	< 0.001
Hips (cm)	107.6 ± 12.5	109.2 ± 12	106.9 ± 12.7	0.002
Systolic BP (mmHg)	126.1 ± 16.5	126.5 ± 15.9	126.0 ± 16.7	0.60
Diastolic BP (mmHg)	76.9 ± 10.1	76.8 ± 10.2	77.0 ± 10	0.67
**Risk Factors**				
Age (> 50 years)	991 (78.0)	273 (65.5)	718 (84.2)	< 0.001
Family history of T2DM	781 (61.5)	297 (71.2)	484 (56.7)	< 0.001
Family history of Osteoporosis	122 (9.6)	42 (10.1)	80 (9.4)	0.38
Family history of Arthritis	75 (5.9)	23 (5.5)	52 (6.1)	0.39
Thyroid Disease	98 (7.7)	34 (8.2)	64 (7.5)	0.38
Barium test (last 2 weeks)	119 (9.4)	33 (7.9)	86 (10.1)	0.13
Scoliosis of Spine	106 (8.3)	28 (6.7)	78 (9.1)	0.08
Kyphosis	50 (3.9)	12 (2.9)	38 (4.5)	0.11
Lost height (2 years)	112 (8.8)	28 (6.7)	84 (9.8)	0.05
Fracture (last 5 years)	134 (10.6)	35 (8.4)	99 (11.6)	0.05
**Biochemical**				
Glucose (mmol/l)	6.9 (5.7,10.2)	7.5 (6,11)	6.7 (5.6,9.9)	0.002
Total Cholesterol (mmol/l)	5.0 ± 1.1	5.1 ± 1.2	5.0 ± 1.1	0.90
Triglycerides (mmol/l)	1.6 (1.2,2.3)	1.7 (1.3,2.4)	1.6 (1.2,2.2)	< 0.001
HDL-Cholesterol (mmol/l)	1.1 ± 0.4	1.1 ± 0.3	1.2 ± 0.4	0.17
Calcium (mmol/l)	2.3 ± 0.3	2.3 ± 0.3	2.3 ± 0.3	0.74
Albumin (g/l)	38.7 ± 6.6	38.5 ± 6.3	38.8 ± 6.7	0.87
SPISE	4.5 ± 1.3	4.3 ± 1.2	4.6 ± 1.3	< 0.001

*Note* Data was presented as mean ± SD and median (Quartile 1, Quartile 3) for normal and non-normal continuous variables and frequency (%) for categorical variables. Overweight meant BMI > 25 and < 30 kg/m2, and obese meant BMI ≥ 30 kg/m2. Independent samples T-test and Mann-Whitney U-test for normal and non-normal variables were used to compare groups; a denotes % among females. A p-value < 0.05 was considered significant

### Bivariate correlation of SPISE with other measured parameters

Bivariate associations between the SPISE index and other parameters were presented in Table 2. Significant inverse correlations between the SPISE index and systolic BP (*r* = -0.24, *p* < 0.001), diastolic BP (*r* = -0.16, *p* < 0.001), and glucose (*r* = -0.35, *p* < 0.001) were found in all participants and similar associations were found in both groups. A significant inverse correlation was found between the SPISE index and T-score (spine) (*r* = -0.21, *p* < 0.001) in all participants and even after stratification according to BMD status.

**Table 2:** Association of SPISE with other measured parameters in the study groups

	All		Normal BMD		Low BMD	
*N*	1270		417		853	
	*r*	*p*	*r*	*p*	*r*	*p*
Age (years)	-0.03	0.31	-0.19	< 0.001	0.02	0.65
Systolic BP (mmHg)	-0.24	< 0.001	-0.26	< 0.001	-0.23	< 0.001
Diastolic BP (mmHg)	-0.16	< 0.001	-0.17	< 0.001	-0.16	< 0.001
Glucose (mmol/l)	-0.35	< 0.001	-0.31	< 0.001	-0.36	< 0.001
Calcium (mmol/l)	0.05	0.15	0.02	0.79	0.07	0.101
Albumin (g/l)	0.04	0.28	0.16	0.004	-0.02	0.57
T-Score Spine	-0.21	< 0.001	-0.11	0.03	-0.25	< 0.001

*Note* Data was presented as bivariate correlation coefficients and the associated p-values. Pearson’s or Spearman’s correlation coefficients were presented for normal and non-normal variables. A *p* < 0.05 was considered significant

### Association between BMD and SPISE index (multivariate analysis)

A multivariate linear regression analysis was done using T-score as the dependent variable and SPISE score as the independent variable following these models: unadjusted model, model a (adjusted for age), model b (adjusted for age and family history of T2DM and model c (adjusted for age, family history of T2DM, time since menopause, whether/not lost height in the past 2 years, and whether/not having history of fracture in the last 5 years) and the results were presented as Table 3. A significant inverse bivariate correlation between BMD and SPISE was found in all participants (β = -0.22, *p* < 0.001), and it remained significant even after stratification between genders. A significant inverse association between BMD and SPISE index was also seen after adjustments in both normal BMD as well as low BMD group (β = -0.10, *p* = 0.02; and β = -0.15, *p* < 0.001 respectively). A separate regression model with T-score as the dependent variable and BMI, HDL and triglycerides (used for the calculation of SPISE) as independent variables was done and presented in supplementary table S1.

**Table 3:** Multivariate linear regression analysis between BMD and SPISE

		All		Females		Males	
		β (95% CI)	*p*	β (95% CI)	*p*	β (95% CI)	*p*
All (*N* = 1270)	Unadjusted	-0.21 (-0.27, -0.16)	< 0.001	-0.23 (-0.28, -0.17)	< 0.001	-0.17 (-0.38, -0.04)	0.01
	Model a	-0.22 (-0.27, -0.17)	< 0.001	-0.24 (-0.28, -0.17)	< 0.001	-0.2 (-0.41, -0.07)	0.004
	Model b	-0.20 (-0.26, -0.16)	< 0.001	-0.22 (-0.27, -0.16)	< 0.001	-0.21 (-0.43, -0.08)	0.004
	Model c	-0.22 (-0.27, -0.17)	< 0.001	-0.23 (-0.27, -0.17)	< 0.001	-0.21 (-0.43, -0.01)	0.005
Normal BMD (*N* = 417)	Unadjusted	-0.08 (-0.14, -0.01)	0.03	-0.07 (-0.15, 0.01)	0.08	-0.13 (-0.28, 0.01)	0.07
	Model a	-0.09 (-0.16, -0.02)	0.02	-0.08 (-0.16, -0.01)	0.03	-0.15 (-0.30, 0.01)	0.05
	Model b	-0.08 (-0.15, -0.01)	0.02	-0.08 (-0.16, -0.01)	0.04	-0.16 (-0.31, -0.01)	0.05
	Model c	-0.10 (-0.17, -0.02)	0.02	-0.09 (-0.17, -0.01)	0.02	-0.15 (-0.31, 0.01)	0.06
Low BMD (*N* = 853)	Unadjusted	-0.15 (-0.18, -0.11)	< 0.001	-0.15 (-0.19, -0.12)	< 0.001	-0.06 (-0.20, 0.07)	0.35
	Model a	-0.15 (-0.18, -0.11)	< 0.001	-0.15 (-0.19, -0.12)	< 0.001	-0.05 (-0.18, 0.09)	0.49
	Model b	-0.14 (-0.18, -0.10)	< 0.001	-0.15 (-0.19, -0.11)	< 0.001	-0.05 (-0.19, 0.09)	0.46
	Model c	-0.15 (-0.19, -0.11)	< 0.001	-0.15 (-0.19, -0.11)	< 0.001	-0.05 (-0.19, 0.09)	0.48

*Note* Data was presented as standardized β-coefficients (95% confidence intervals, CI). Unadjusted model (SPISE only), model a (adjusted for age), model b (adjusted for age and family history of T2DM), model c (adjusted for age, family history of T2DM, menopausal status in females, lost height in the past 2 years and history of fracture in the last 5 years). A *p* < 0.05 was considered significant

**Fig. 3:**
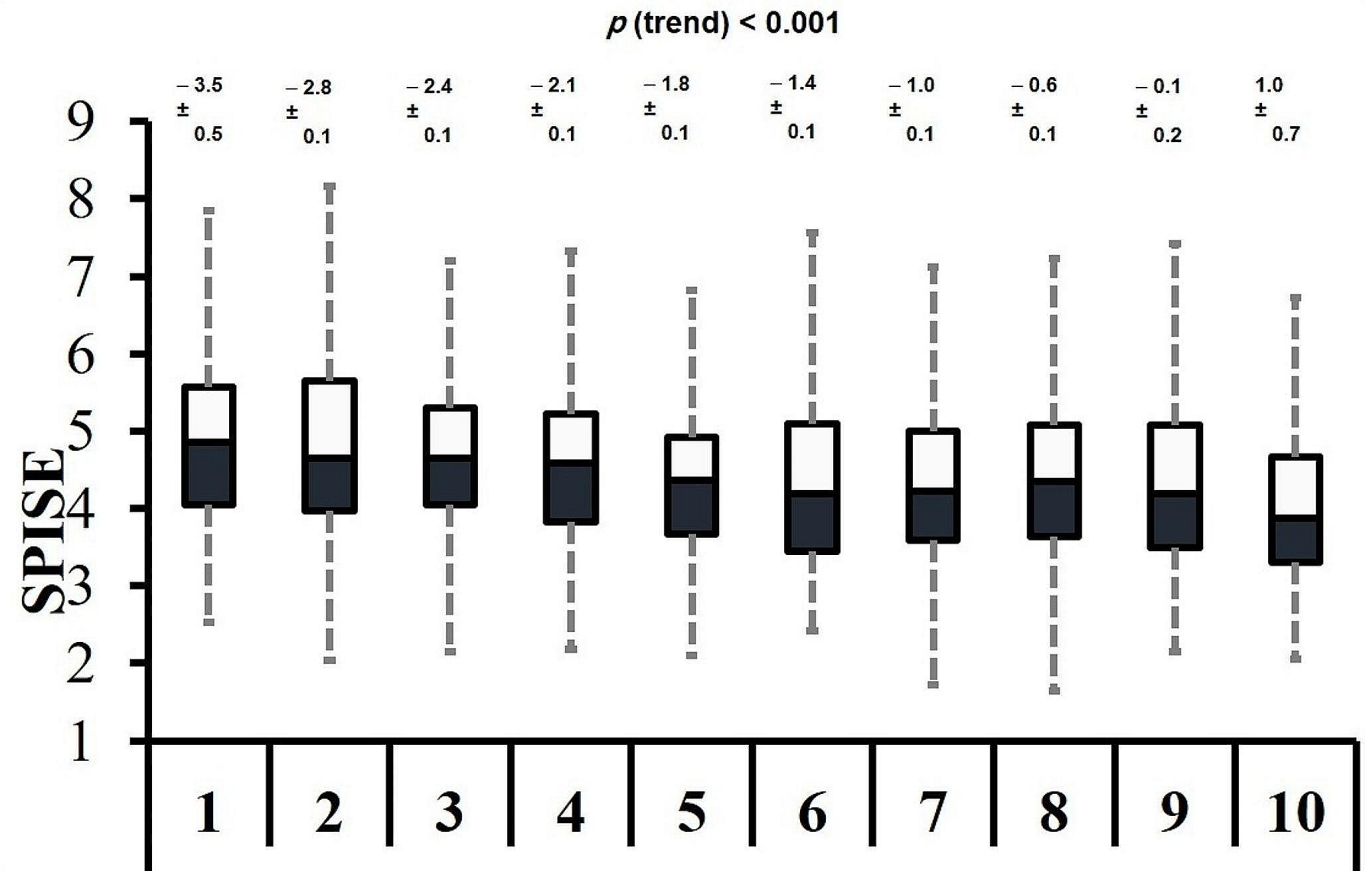
Box plots representing the decreasing overall trend of SPISE values in increasing T-score (spine) deciles

A box plot representing the data of SPISE values in different deciles of T-score (spine) was presented as Fig. 3. A decreasing overall trend in SPISE values was seen with increasing deciles of T-score (spine).

### Significant predictors of BMD (stepwise regression analysis)

A stepwise regression analysis was applied using BMD (T-score) as the dependent variable and the following independent variables: SPISE, age, family history of T2DM, time since menopause, whether/not lost height in the past 2 years, and whether/not having a history of fracture in the last 5 years and the results were presented as Table 4. Covariates were based on risk factors of bone loss that were significantly different between the normal BMD and low BMD groups (Table 1). When stratified between normal BMD and low BMD groups, SPISE and age were the significant predictors of T-score in the normal BMD group; while SPISE, age, family history of T2DM and history of fracture were the significant predictors of T-score in low BMD group. Overall when the data from all participants were considered, in the fully adjusted model, SPISE, family history of T2DM and history of fracture predicted 17.4% of the total variance in the T-score (spine).

**Table 4:** Stepwise linear regression analysis depicting significant predictors of T-score (spine)

Significant predictors	β ± SE	95% CI		*p*	Adjusted *R*^2^
		lower	upper		
**Normal BMD (417)**					
SPISE	-0.08 ± 0	-0.16	-0.01	0.034	0.025
Age	-0.01 ± 0	-0.03	0.00	0.010	
**Low BMD (853)**					
SPISE	-0.15 ± 0	-0.19	-0.11	< 0.001	0.121
Age	-0.02 ± 0	-0.03	-0.01	< 0.001	
Family history of T2DM	0.13 ± 0.1	0.03	0.24	0.012	
Fracture (last 5 years)	-0.21 ± 0.1	-0.37	-0.04	0.015	
**All (1270)**					
SPISE	-0.22 ± 0	-0.27	-0.17	< 0.001	0.174
Family history of T2DM	0.3 ± 0.1	0.16	0.44	< 0.001	
Fracture (last 5 years)	-0.31 ± 0.1	-0.53	-0.10	0.004	

*Note* Data was presented as β-coefficients ± Standard errors and its 95% confidence intervals. BMD (T-score) was used as the dependent variable and the following independent variables were used: SPISE, age, family history of T2DM, time since menopause, whether/not lost height in the past 2 years, and whether/not having a history of fracture in the last 5 years. The table only shows the fully adjusted models. A *p* < 0.05 was considered significant

## Discussion

The present study explored the relationship between the SPISE index, a composite measure indicative of insulin sensitivity, and bone mineral density in an adult population and is the first to observe a significant inverse correlation between BMD (spine) and SPISE in a homogenous adult Saudi Arab ethnic group. The multivariate regression analysis revealed this inverse association was significant even after adjusting for relevant covariates such as age, family history of T2DM, menopausal status, and history of fractures. This suggests that the SPISE index, proposed as a marker for insulin sensitivity, may have wider implications that influence other systems such as skeletal health. The inverse correlation observed between the SPISE index and BMD raises relevant questions about potential underlying mechanisms and the intricate crosstalk between metabolism and bone health in the studied population.

With the advent of urbanization and an ageing society, T2DM and osteoporosis are becoming more prevalent, and current findings for the association between the two have been consistent [34, 35]. Impaired glucose metabolism can affect bone health, and T2DM has historically been linked to an increased risk of fractures and poor bone health [36, 37]. Besides, since T2DM might affect osteoblasts and osteoclasts, it is possible that this T2DM-induced imbalance between the two bone-remodeling cells could result in osteoporosis [38, 39]. The mechanism of the action of circulating insulin levels on bone cells has not been fully elucidated yet. Still, the presence of insulin receptors on both osteoblasts and osteoclasts suggests the regulation of insulin signalling in bone formation and resorption processes [40, 41].

In this study, a significant inverse correlation between SPISE and BMD (spine) was observed when overall data was taken into consideration, and significantly higher average SPISE index values were observed in the group with low BMD (spine) compared with the group having normal BMD (4.6 vs. 4.3, *p* < 0.001). This observation is in line with the findings that individuals with T2DM have higher BMD values than those without T2DM [42, 43]. Furthermore, in a study of 93,676 postmenopausal women [44], those with T2DM had greater spine and hip BMD values. This observed inverse relationship may partially be explained by obesity’s role which is considered a protective factor for osteoporosis [45–47]. Furthermore, higher BMI and waist circumferences are associated with higher 17 β-estradiol levels which are known to improve BMD by inhibiting bone resorption and favoring bone formation [48]. Also, insulin-growth factor (IGF)-1 receptors have been shown to interact with hyperglycemia to improve BMD by encouraging osteoblast development [49]. Other investigations pointed out that although BMD is higher in T2DM, the higher BMD encompasses an increase in aortic calcifications [50], osteophytes [51], and glycation end-products [52], making the bones more fragile, partially explaining the increased fracture rate in T2DM [53]. The relationship between BMD and IR has also been reported to be significantly affected by sex hormones in maintaining bone health [54, 55]. This sexual dimorphism was not observed in the present study.

When the entire group was considered, the adjusted model in the analysis consistently generated significant associations for the following groups: normal BMD (β = -0.10, *p* = 0.02), low BMD (β = -0.15, *p* < 0.001), females (β = -0.23, *p* < 0.001), and males (β = -0.21, *p* = 0.005). These results suggest SPISE index exhibits an inverse correlation with BMD in various groups at varying magnitudes. The significant associations in both males and females and both normal and low BMD groups support the overall relevance of the SPISE index in influencing bone health. The SPISE index, which incorporates BMI, HDL-cholesterol, and triglycerides, provides a non-invasive and cost-effective measure for assessing insulin sensitivity and given the observed associations with the BMD, it may also offer valuable insights into the complex interplay between metabolic factors and bone health.

In our study, we used stepwise regression analysis to identify significant predictors of BMD and the analysis suggested SPISE, family history of T2DM, and history of fractures together explained 17.4% of the total variances perceived in the T-score (spine). In particular, as discussed, SPISE demonstrated a statistically significant inverse correlation with BMD (β = -0.22, *p* < 0.001), indicating that decreased insulin sensitivity may be associated with a reduction in bone density. An additional significant predictor was a family history of T2DM, which demonstrates the genetic and metabolic interaction between diabetes and bone health [56–59]. Moreover, the lowest BMD was substantially associated with a fracture history, which is consistent with the well-established notion that previous fractures are a reliable predictor of osteoporosis and fracture risk in the future [60, 61]. Although lifestyle factors such as smoking, physical activity, and diet were not documented, the observed significant associations highlight the predictive value of metabolic health indicators concerning bone health. The results of the stepwise analysis provided in this study signify the complex interplay between metabolic and hereditary elements that impact bone health.

Lastly, it is important to highlight that the statistical association between SPISE and T-score was observed only when hyperglycemia was present (supplementary table S2), and whether this association was ‘real’ or just driven by confounders cannot be answered fully in the present study, given its cross-sectional design. The present findings are somewhat similar to a recent observational study using data from NHANES involving more than 5292 participants which showed that the association between BMD T-score and HOMA-β, a measure of insulin sensitivity, was largely influenced by the individual’s insulin resistance status [62]. Altered glucose metabolism such as hyperglycemia and insulin resistance are known to induce the formation and accumulation of advanced glycation end-products (AGEs) which has a negative effect on bone health [63]. Elevated glucose levels and AGEs stimulate the expression of sclerostin, a negative regulator of bone formation [64], and consequently, osteoprotegerin, a known inhibitor of bone resorption which has been observed to be positively associated with insulin resistance in postmenopausal women [65], with higher levels reported among T2DM as compared to non-T2DM individuals [66]. This may partially explain why the elicited association was only significant in the presence of hyperglycemia, as AGEs may exert their negative effects on skeletal health (including the progression of diabetic complications) only when it has started to accumulate within tissues of insulin-resistant and chronically hyperglycemic individuals [67].

The authors acknowledge some limitations and hence the results should be interpreted with caution. First, the study’s cross-sectional design was unable to infer causality in the reported associations in this study. Second, the effects of hormonal changes on bone metabolism due to ageing were not examined in this study’s reported associations. Additionally, we acknowledge that the absence of data on some lifestyle factors like physical activity, diet, etc. is a limitation. Glycemic indices such as insulin and HOMA-IR, as well as medications that may affect bone and/or glucose metabolism were not evaluated. Nevertheless, the influence of age and sex was controlled in the regression analysis. Despite the mentioned caveats, the merits of this research include a relatively large population, the use of DXA and the use of an understudied index for assessing insulin sensitivity that integrates the influence of adiposity in terms of BMI. Meanwhile, the present findings may be clinically useful in assessing the bone health of T2DM patients where healthcare resources are scarce and BMD assessment is not possible, given that SPISE can be calculated in the absence of known glycemic indices. Our study was conducted on a cohort of predominantly Arab adult females with a high prevalence of overweight and obesity. The specific demographic and clinical characteristics of our cohort should be considered when generalizing our findings. While the significant associations observed between SPISE and BMD suggest a robust link applicable to populations with similar metabolic and demographic profiles, caution should be exercised in extending these results to different ethnic or age groups without further validation. Future research should aim to replicate our study in diverse populations to confirm the generalizability of our findings.

## Conclusion

In conclusion, our cross-sectional observational study offers valuable insights into the intricate relationship between the SPISE index, a composite marker of metabolic health, and BMD in a Saudi adult population. The significant negative association between the SPISE index and BMD, even after multiple adjustments for various confounding factors, highlights the potential relevance of metabolic factors in influencing bone health. These results have several implications for clinical practice beyond the studied population. Given the global rise in both metabolic disorders and osteoporosis, healthcare providers may consider integrating metabolic health assessments, such as the SPISE index, into routine evaluations for patients at risk of osteoporosis. Further exploration of these associations in longitudinal studies and intervention trials could provide valuable insights into the complex relationship between metabolic parameters and bone density, which may help in targeted interventions and preventive strategies for individuals at risk of osteoporosis.

### Electronic supplementary material

Below is the link to the electronic supplementary material.

Supplementary Material 1

## Data Availability

The data presented in this study are available upon request from the corresponding author.
