# Supplementary material for: The single point insulin sensitivity estimator (SPISE) is associated with bone health in Arab adults
Source: Aging Clin Exp Res. 2024 Jun 21;36(1):136. doi: 10.1007/s40520-024-02789-5 (PMC11192813; doi:10.1007/s40520-024-02789-5)
Supplement: Supplementary file 1 — Supplementary Material 1 [file 40520_2024_2789_MOESM1_ESM.docx]

**Appendix1: Supplementary Tables**

**Table S1:** Multivariate linear regression analysis between T-score and BMI, HDL and triglycerides

|  | | **All (1270)** | | **Females (1067)** | | **Males (203)** | | **Normal BMD (417)** | | **Low BMD (853)** | |
| --- | --- | --- | --- | --- | --- | --- | --- | --- | --- | --- | --- |
|  |  | **β (95% CI)** | ***p*** | **β (95% CI)** | ***p*** | **β (95% CI)** | ***p*** | **β (95% CI)** | ***p*** | **β (95% CI)** | ***p*** |
| BMI | Unadjusted | 0.03 (0.02, 0.05) | <0.001 | 0.04 (0.03, 0.05) | <0.001 | 0.05 (0.01, 0.09) | 0.009 | 0.01 (-0.01, 0.02) | 0.327 | 0.03 (0.02, 0.04) | <0.001 |
|  | Model a | 0.03 (0.02, 0.04) | <0.001 | 0.04 (0.03, 0.05) | <0.001 | 0.05 (0.01, 0.09) | 0.011 | 0.01 (-0.01, 0.00) | 0.308 | 0.03 (0.02, 0.03) | <0.001 |
|  | Model b | 0.03 (0.02, 0.04) | <0.001 | 0.04 (0.03, 0.05) | <0.001 | 0.06 (0.01, 0.09) | 0.008 | 0.01 (-0.01, 0.02) | 0.328 | 0.03 (0.02, 0.03) | <0.001 |
|  | Model c | 0.04 (0.03, 0.05) | <0.001 | 0.04 (0.03, 0.05) | <0.001 | 0.05 (0.01, 0.10) | 0.011 | 0.01 (-0.00, 0.03) | 0.072 | 0.03 (0.02, 0.04) | <0.001 |
| HDL | Unadjusted | -0.15 (-0.34, 0.05) | 0.141 | -0.11 (-0.06, 0.48) | 0.818 | -0.07 (-0.27, 0.13) | 0.479 | -0.25 (-0.48, -0.01) | 0.038 | 0.05 (-0.09, 0.18) | 0.512 |
|  | Model a | -0.22 (-0.41, -0.04) | 0.021 | -0.11 (-0.06, -0.04) | 0.241 | -0.23 (-0.80, 0.34) | 0.429 | -0.28 (-0.51, -0.04) | 0.021 | 0.03 (-0.10, 0.16) | 0.677 |
|  | Model b | -0.20 (-0.39, -0.02) | 0.033 | -0.11 (-0.29, 0.08) | 0.267 | -0.23 (-0.81, 0.34) | 0.425 | -0.26 (-0.49, -0.03) | 0.031 | 0.03 (-0.10, 0.16) | 0.668 |
|  | Model c | -0.15 (-0.33, 0.03) | 0.104 | -0.12 (-0.30, 0.07) | 0.217 | -0.24 (-0.81, 0.34) | 0.418 | -0.25 (-0.48, -0.12) | 0.036 | 0.04 (-0.09, 0.17) | 0.544 |
| Triglycerides | Unadjusted | 0.88 (0.53, 1.23) | <0.001 | 0.54 (0.16, 0.92) | 0.006 | 0.75 (-0.11, 1.61) | 0.086 | 0.38 (0.01, 0.76) | 0.047 | 0.43 (0.17, 0.69) | 0.001 |
|  | Model a | 0.96 (0.62, 1.29) | <0.001 | 0.55 (0.19, 0.92) | 0.003 | 0.89 (0.03, 1.75) | 0.043 | 0.44 (0.05, 0.82) | 0.026 | 0.42 (0.17, 0.68) | 0.001 |
|  | Model b | 0.88 (0.54, 1.21) | <0.001 | 0.51 (0.14, 0.86) | 0.006 | 0.89 (0.03, 1.76) | 0.042 | 0.42 (0.04, 0.81) | 0.031 | 0.38 (0.13, 0.64) | 0.003 |
|  | Model c | 0.62 (0.29, 0.94) | <0.001 | 0.51 (0.16, 0.87) | 0.005 | 0.86 (-0.03, 1.75) | 0.057 | 0.33 (-0.06, 0.72) | 0.093 | 0.32 (0.07, 0.57) | 0.014 |

**Note:** Data was presented as standardized β-coefficients (95% confidence intervals, CI). Unadjusted model (BMI, HDL, and triglycerides only for each model), model a (adjusted for age), model b (adjusted for age and family history of T2DM), model c (adjusted for age, family history of T2DM, menopausal status in females, lost height in the past 2 years and history of fracture in the last 5 years). A p<0.05 was considered significant.

**Table S2:** Multivariate linear regression analysis between BMD and SPISE in presence and absence of hyperglycemia.

|  | | **All** | | **Females** | | **Males** | |
| --- | --- | --- | --- | --- | --- | --- | --- |
|  |  | **β (95% CI)** | ***p*** | **β (95% CI)** | ***p*** | **β (95% CI)** | ***p*** |
| All  (N=1270) | Unadjusted | -0.21 (-0.27, -0.16) | <0.001 | -0.23 (-0.28, -0.17) | <0.001 | -0.17 (-0.38, -0.04) | 0.01 |
|  | Model a | -0.22 (-0.27, -0.17) | <0.001 | -0.24 (-0.28, -0.17) | <0.001 | -0.2 (-0.41, -0.07) | 0.004 |
|  | Model b | -0.2 (-0.26, -0.16) | <0.001 | -0.22 (-0.27, -0.16) | <0.001 | -0.21 (-0.43, -0.08) | 0.004 |
|  | Model c | -0.22 (-0.27, -0.17) | <0.001 | -0.23 (-0.27, -0.17) | <0.001 | -0.21 (-0.43, -0.01) | 0.005 |
| Healthy  (N=173) | Unadjusted | -0.07 (-0.13, 0.05) | 0.36 | -0.08 (-0.15, 0.05) | 0.33 | -0.11 (-0.25, 0.14) | 0.57 |
|  | Model a | -0.21 (-0.17, 0.01) | 0.09 | -0.13 (-0.18, 0.02) | 0.12 | -0.15 (-0.30, 0.16) | 0.54 |
|  | Model b | -0.17 (-0.17, 0.02) | 0.11 | -0.13 (-0.18, 0.02) | 0.13 | -0.13 (-0.31, 0.18) | 0.60 |
|  | Model c | -0.15 (-0.18, 0.01) | 0.06 | -0.15 (-0.20, 0.01) | 0.08 | -0.09 (-0.30, 0.22) | 0.74 |
| Low BMD  alone  (N=469) | Unadjusted | -0.06 (-0.19, 0.07) | 0.36 | -0.08 (-0.22, 0.08) | 0.34 | -0.15 (-0.40, 0.08) | 0.18 |
|  | Model a | -0.06 (-0.18, 0.07) | 0.32 | -0.08 (-0.03, 0.01) | 0.29 | -0.14 (-0.39, 0.09) | 0.23 |
|  | Model b | -0.06 (-0.19, 0.07) | 0.39 | -0.07 (-0.22, 0.08) | 0.35 | -0.14 (-0.39, 0.09) | 0.23 |
|  | Model c | -0.09 (-0.20, 0.04) | 0.18 | -0.09 (-0.24, 0.07) | 0.27 | -0.13 (-0.39, 0.12) | 0.28 |
| Hyperglycemia alone  (N=244) | Unadjusted | -0.29 (-0.22, -0.12) | <0.001 | -0.31 (-0.22, -0.12) | <0.001 | -0.06 (-0.24, 0.17) | 0.74 |
|  | Model a | -0.3 (-0.21, -0.12) | <0.001 | -0.31 (-0.22, -0.12) | <0.001 | -0.09 (-0.26, 0.17) | 0.66 |
|  | Model b | -0.28 (-0.21, -0.11) | <0.001 | -0.3 (-0.21, -0.12) | <0.001 | -0.11 (-0.28, 0.16) | 0.58 |
|  | Model c | -0.28 (-0.21, -0.11) | <0.001 | -0.29 (-0.21, -0.11) | <0.001 | -0.11 (-0.29, 0.17) | 0.59 |
| Low BMD + Hyperglycemia  (N=384) | Unadjusted | -0.14 (-0.19, -0.03) | 0.005 | -0.17 (-0.23, -0.05) | 0.002 | -0.18 (-0.34, 0.06) | 0.17 |
|  | Model a | -0.11 (-0.17, -0.01) | 0.03 | -0.15 (-0.21, -0.03) | 0.008 | -0.08 (-0.28, 0.16) | 0.59 |
|  | Model b | -0.11 (-0.17, -0.01) | 0.03 | -0.15 (-0.21, -0.03) | 0.009 | -0.07 (-0.28, 0.17) | 0.62 |
|  | Model c | -0.14 (-0.19, -0.03) | 0.01 | -0.15 (-0.21, -0.04) | 0.006 | -0.07 (-0.29, 0.17) | 0.61 |

**Note:** Data was presented as standardized β-coefficients (95% confidence intervals, CI). Healthy denotes individuals with normoglycemia and normal BMD. Unadjusted model (SPISE only), model a (adjusted for age), model b (adjusted for age and family history of T2DM), model c (adjusted for age, family history of T2DM, menopausal status in females, lost height in the past 2 years and history of fracture in the last 5 years). A p<0.05 was considered significant.

**Appendix2: Supplementary Figures**


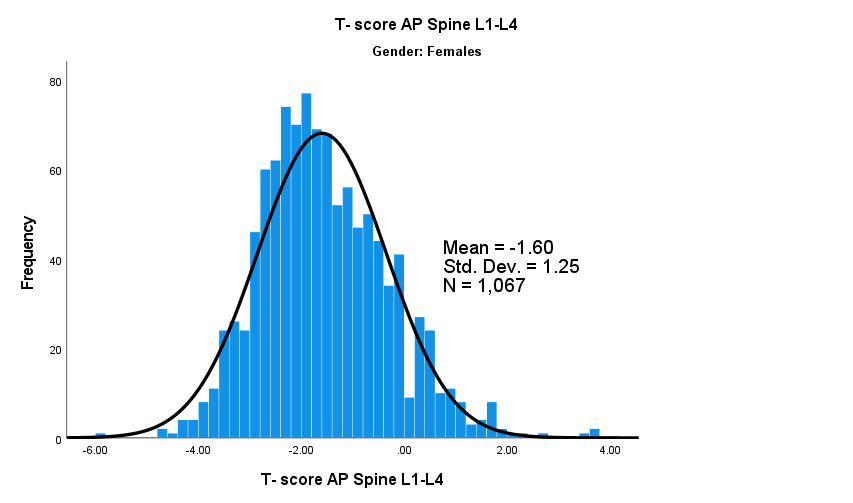

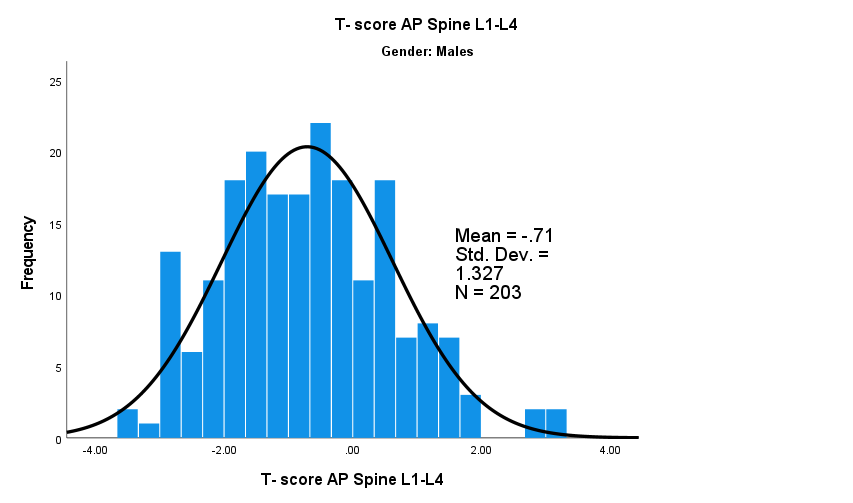

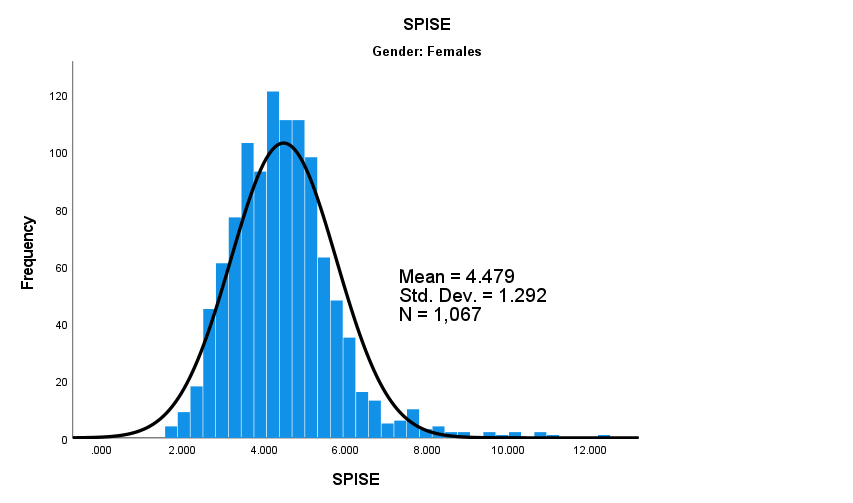

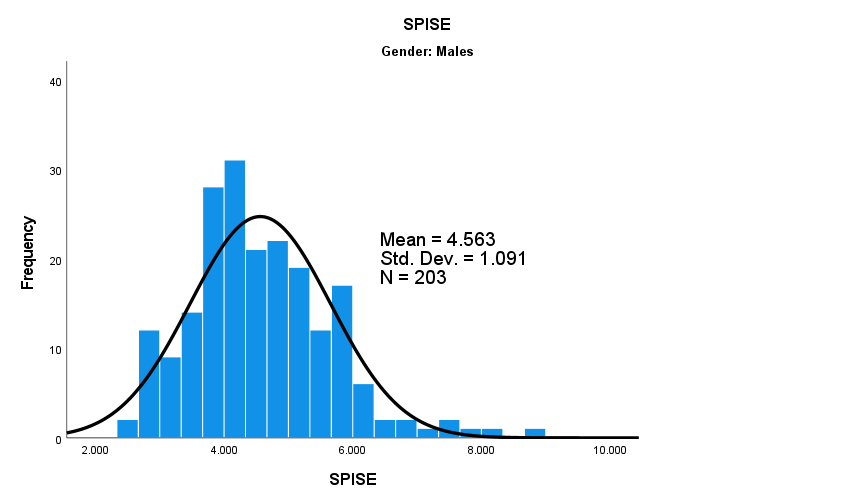


**Supplementary figure S1:** Histograms depicting the distribution of T-score and SPISE index in females and males in the study population.


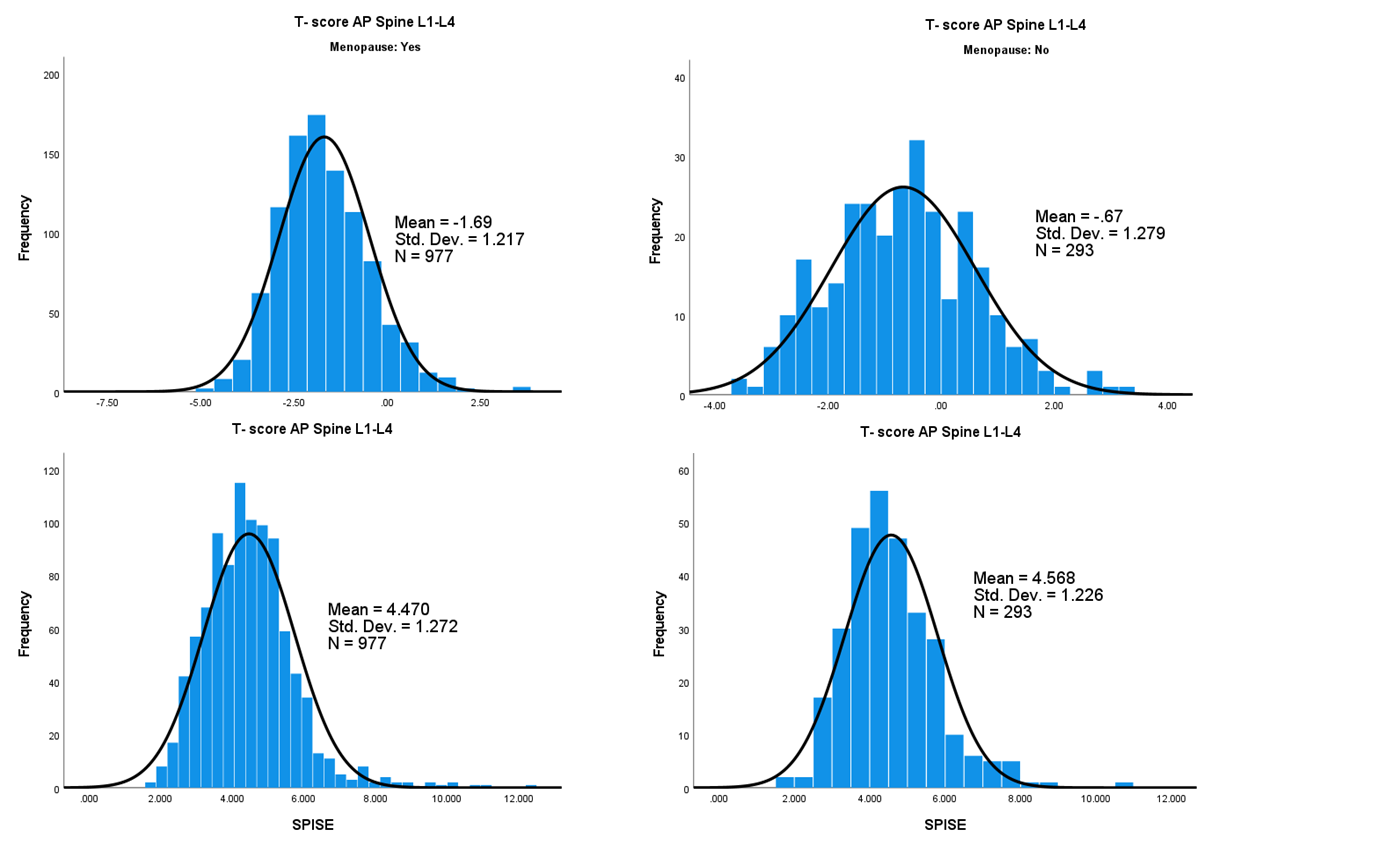


**Supplementary figure S2:** Histograms depicting the distribution of T-score and SPISE index in post and pre-menopausal women.
